# Traumatic Stress, Attachment Style, and Health Outcomes in Cardiac Rehabilitation Patients

**DOI:** 10.3389/fpsyg.2020.00075

**Published:** 2020-01-28

**Authors:** Adam Heenan, Paul S. Greenman, Vanessa Tassé, Fotini Zachariades, Heather Tulloch

**Affiliations:** ^1^Division of Cardiac Prevention and Rehabilitation, University of Ottawa Heart Institute, Ottawa, ON, Canada; ^2^Département de Psychoéducation et de Psychologie, Universite du Québec en Outaouais, Gatineau, QC, Canada; ^3^Institut du Savoir Montfort, Ottawa, ON, Canada; ^4^Children’s Hospital of Eastern Ontario, Ottawa, ON, Canada; ^5^Department of Medicine, Faculty of Medicine, University of Ottawa, Ottawa, ON, Canada; ^6^School of Psychology, University of Ottawa, Ottawa, ON, Canada

**Keywords:** cardiovascular disease, attachment anxiety, traumatic stress, health outcomes, attachment avoidance

## Abstract

**Objective:**

Research on psychosocial risk factors in cardiovascular disease (CVD) has identified traumatic stress and attachment style as independent risk factors for the development of CVD and poor prognosis for those with established CVD. Exploring the interrelationships between these variables will inform psychosocial risk factor modeling and potential avenues for intervention. Therefore, the hypothesis that attachment style is related to health outcomes among CR patients and that traumatic stress mediates this relationship was tested.

**Methods:**

Patients in a cardiac rehabilitation program (*n* = 201) completed validated self-report measures of traumatic stress and attachment style at baseline (program intake). Health outcomes were assessed at baseline and 3 months, including anxiety, depression, quality of life, fasting blood glucose, glycated hemoglobin (HbA1c), and cholesterol (HDL ratio). Multivariate structural equation modeling was used to fit the data.

**Results:**

Of the 201 participants, 42 (21%) had trauma scores indicating the probable presence of posttraumatic stress disorder. Via greater levels of traumatic stress, greater attachment anxiety at baseline was indirectly related to greater anxiety, depression, fasting blood glucose, and HbA1c, and poorer physical and mental quality of life. There were no significant indirect effects on HDL ratios.

**Conclusion:**

Greater attachment anxiety predicted greater traumatic stress; this, in turn, predicted poorer health outcomes. Screening and treatment for these constructs in CVD patients is warranted.

## Introduction

Cardiovascular disease (CVD) includes a host of different disorders of the heart and blood vessels, such as coronary artery disease and hypertension, and symptoms typically manifest only after the disease has progressed to an advanced stage (Perk et al., 2012). Prevalence rates of CVD in the United States (Mozaffarian et al., 2015) and Canada (Government of Canada, 2019) are very high (6.2 and 7.8%, respectively), especially for an ostensibly preventable disease (Mozaffarian et al., 2015), and these rates are associated with a significant societal financial burden (Trogdon et al., 2007; Mozaffarian et al., 2015). As the population of people with CVD in developed countries continues to increase, so does the need for preventative medicine (Yazdanyar and Newman, 2009).

The promotion of health behavior change is central to CVD prevention efforts. These changes focus on reducing or moderating specific risk factors; these can be behavioral in nature, such as managing weight loss, quitting smoking, or eating healthier (Mozaffarian et al., 2015), they can be physiological, such as targeting specific CVD markers like hypertension, high cholesterol, or lack of exercise (Lee et al., 2012), or, these risk factors can psychological, such as elevated anxiety and depression (Rothenbacher et al., 2007; Whalley et al., 2014; Blumenthal et al., 2016), insecure attachment (Maunder and Hunter, 2001), and lower levels of psychological well-being (Boehm and Kubzansky, 2012).

Interest in attenuating psychological risk factors has grown in recent years, as some targeted psychosocial interventions have proven effective in reducing cardiac events (Whalley et al., 2014; Blumenthal et al., 2016; Richards et al., 2018), which is positive, as individuals with CVD are known to have elevated rates of anxiety (Frasure-Smith and Lespérance, 2008; Bunevicius et al., 2013) and depression (Frasure-Smith and Lespérance, 2008; Lichtman et al., 2008). Stress and depression directly contribute to poorer cardiovascular health by increasing blood pressure (Rothenbacher et al., 2007), vascular inflammation, and endothelial dysfunction (Williams and Steptoe, 2007). Indirectly, greater anxiety and depression promote other unhealthy behaviors (e.g., sedentary behavior, smoking) that exacerbate CVD risk.

### Insecure Attachment as a Psychosocial Risk Factor for CVD

One potential psychosocial risk factor for CVD that has not yet been fully explored is insecure attachment, a construct that has been associated with increased prevalence of illness and disease (Maunder and Hunter, 2001; McWilliams and Bailey, 2010; Pietromonaco et al., 2013; Pietromonaco and Collins, 2017). Attachment was first described in the literature over four decades ago; it is defined as the tendency for human beings to develop strong emotional bonds with significant people in their lives, referred to as “attachment figures,” as a principal means of emotion regulation in the face of stress or potential danger (Bowlby, 1958, 2005; Ainsworth and Bell, 1970). Secure attachment refers to the tendency to seek out emotional support and comfort from an attachment figure in situations of stress or emotional pain. Securely attached individuals perceive others as safe and trustworthy (Bowlby, 1980); thus, they are able to receive comfort and reassurance when they need it to reduce stress. Insecure attachment, however, is characterized by the tendency to either avoid seeking out emotional support and closeness from others (i.e., avoidant attachment) or exist in a near-constant state of reassurance-seeking that often leaves one feeling distressed and vulnerable, rather can calm and safe (i.e., anxious attachment; Bartholomew and Horowitz, 1991; Simpson and Rholes, 2017).

Instead of deriving reassurance and comfort from their relationships with attachment figures, people who are insecurely attached are more likely to worry and to feel uneasy about these relationships. This is highly stressful and is thought to contribute to the onset and course of chronic disease (Maunder and Hunter, 2001; McWilliams and Bailey, 2010; Pietromonaco et al., 2013; Pietromonaco and Collins, 2017). More specifically, the theorized mechanisms by which insecure attachment relates to increased disease risk include higher cortisol levels during times of stress (Luecken, 1998; Quirin et al., 2008; Pierrehumbert et al., 2012), augmented blood pressure during social interactions (Gallo and Matthews, 2006), and heavier reliance on external regulators for emotions, including alcohol and tobacco (Maunder and Hunter, 2001).

Although there is a known link between a lack of social relationships and increased mortality in general (Cerhan and Wallace, 1997; Holt-Lunstad et al., 2010; Barger, 2013), only a handful of studies have examined the impact of attachment style on heart health (Vilchinsky et al., 2010; Pietromonaco et al., 2013; Peleg et al., 2017; Pietromonaco and Collins, 2017). This may indicate an important gap in the literature, as the studies that have been conducted suggest a connection between insecure attachment and poor health (West et al., 1995; Bohachick et al., 2002; McWilliams and Bailey, 2010; Pietromonaco and Collins, 2017). For example, in one sample of the general population (*N* = 5645), insecure attachment was identified as a risk factor for the development of chronic illness, especially conditions related to the cardiovascular system (McWilliams and Bailey, 2010). Likewise, a follow-up study of heart transplant patients revealed that those with insecure attachment had worse functional outcomes 6 months after the procedure than did patients with secure attachment (Bohachick et al., 2002). A recent investigation found similar results; the researchers found insecure attachment styles were significantly more prevalent among people with coronary artery disease compared to healthy controls (Oladi and Dargahi, 2018).

There are known mechanistic links between attachment and CVD risk factors; for example, inflammation has been identified as a major contributor to the onset of heart disease, and there is evidence that both avoidant and anxious attachment are related to the production of the IL-6 in stressful situations such as conflict discussions between spouses and even CABG surgery (Kidd et al., 2014; Ehrlich, 2019). Despite some anti-inflammatory properties that have been noted, IL-6 is generally considered atherogenic, to the point where some researchers have suggested targeting it as a treatment for atherosclerosis (Reiss et al., 2017). Cardiovascular reactivity is another risk factor that researchers have linked to attachment and the development of CVD. Compared to when doing a cold pressor task alone, securely attached participants had significantly reduced systolic and diastolic blood pressure when their primary attachment figures were present or when they were instructed to think about them (Bourassa et al., 2019). Greater reductions in heart rate variability have also been detected during this stress task among participants with high levels of attachment avoidance (Bryant and Hutanamon, 2018). Once again, greater attachment security was related to less cardiovascular risk, and vice versa. Lastly, attachment insecurity has also been linked to primary hypertension. One group of researchers observed that 88% of hypertensive patients presented as insecurely attached, as compared to 50% in non-clinical populations (Balint et al., 2016).

### Attachment, Psychological Functioning, and the Heart

Insecure attachment throughout the lifespan is also a robust predictor of psychopathology. For example, Stovall-McClough and Dozier (2016) noted in a comprehensive review of the literature that anxious, avoidant, and disorganized (i.e., sometimes anxious, sometimes avoidant) attachment styles detected during childhood play a key role in the development of major depression, anxiety disorders, dissociative disorders, and personality disorders later in life. The findings of more recent studies corroborate these results; insecure attachment (anxious and avoidant) has been linked to major depression comorbid with generalized anxiety disorder (GAD), panic disorder, and PTSD in adults (Huang et al., 2019), whereas fear of rejection by romantic partners and fearful attachment in general has also predicted the presence of mental-health problems among adults (Alonso et al., 2018). Taken together, these findings suggest that the subjective sense of loneliness typical of people with insecure attachment styles might contribute to the onset and course of psychological problems. This makes sense considering that, according to attachment theory, the perceived emotional unavailability of an attachment figure, especially in times of stress, is inherently traumatizing for human beings (Johnson, 2020), with all of the physical and psychological implications that the experience of trauma can have.

Symptoms of traumatic stress can also occur following a cardiac event; in fact, a systematic review of PTSD induced by a cardiac event revealed an average prevalence of about 12% of cardiac samples across 150 studies and noted that pre-existing psychological risk factors were important predictors in developing PTSD in this manner (Vilchinsky et al., 2017). Symptoms of PTSD, generally, include intrusive thoughts or images (flashbacks, nightmares), avoidance of triggering stimuli, emotional numbing, hyperarousal, sleep difficulties, and negative cognitions and mood (American Psychiatric Association, 2013). A diagnosis of PTSD is made when symptoms of traumatic stress are significant enough to disrupt an individual’s functioning or to cause them clinically significant distress. A recent systematic review of 21 studies of attachment and post-traumatic stress in the general population revealed that insecure attachment consistently had mediating and moderating effects on the link between the experience of a traumatic stressor and the onset of symptoms of post-traumatic stress (Barazzone et al., 2019). The more insecurely attached a person is, the more likely he or she appears to develop PTSD in the face of a traumatic event. Potential reasons for this correlation might be that people with insecure attachment perceive a lack of emotional support and are therefore less resilient to trauma. Those with anxious attachment also report greater distress than do those with secure or avoidant attachment, indicating that they may be more focused on, and sensitive to, threatening stimuli.

With respect to cardiovascular functioning, chronic traumatic stress is detrimental to the heart; it is linked to increased heart rate, blood pressure, and substance abuse (Bedi and Arora, 2007; Coughlin, 2011); as well as greater risk of developing CVD (Kubzansky et al., 2007, 2009; Boscarino, 2008) and negative physical and mental health outcomes in patients with CVD (Whitehead et al., 2006; Edmondson and Cohen, 2013; Ginzburg et al., 2016). In one study of over 1000 patients with CVD followed over 7.5 years, exposure to traumatic events throughout the lifespan predicted greater mortality and a greater number of adverse cardiovascular events (Hendrickson et al., 2013). Moreover, the experience of potentially fatal cardiovascular events (e.g., myocardial infarction) has been shown to induce PTSD in some patients (Spindler and Pedersen, 2005; Edmondson et al., 2011, 2012; Edmondson and Cohen, 2013), and PTSD symptoms after such an event, in turn, are linked to subsequent cardiac health problems (Wikman et al., 2008; Edmondson et al., 2011) and all-cause mortality (Edmondson et al., 2011). To our knowledge, no one has yet examined the role of both attachment insecurity and traumatic stress in a CVD population, despite the potential for attachment insecurity to explain some of the well-documented effects of trauma on the heart.

## Objectives

The main objective of this study was to test a model predicting health outcomes measured at 3 months in a CR population using baseline measures of attachment style and traumatic stress. Multivariate SEM was used to test the following hypotheses: (1) insecure attachment (i.e., avoidant or anxious attachment) would be associated with greater traumatic stress symptoms, (2) elevated traumatic stress and insecure attachment would each be independently associated with poorer cardiac-related physical and mental health outcomes, and (3) traumatic stress would mediate the relationship between insecure attachment and poorer physical and mental health outcomes.

## Materials and Methods

### Participants

Patients enrolled in the cardiac rehabilitation program (CR) at the UOHI were assessed at baseline (program intake) and 3 months (end-of-program). The CR program consists of exercise, education, and allied health services (e.g., nurses, physiotherapists, vocational rehabilitation). Patients are automatically referred to CR following discharge from hospital (Tiller et al., 2013). Only those who were unable to speak/read in English or French were excluded from the study. This study was approved by the Ottawa Health Science Network Research Ethics Board.

A power analysis was conducted using the software G^∗^Power 3 (Faul et al., 2007) in order to calculate the minimum sample size required to achieve sufficient power for the statistical analyses involved. With a power level a 0.80 and an alpha of 0.05, it was estimated that a sample size of 200 would be required. This is also in keeping with the minimum suggested sample size for SEM (i.e., *N* = 200), suggested by Kline (2011).

### Procedures

Individuals participating in the CR program at the UOHI were recruited at the patient’s standard intake into the program. A healthcare professional in the prospective participant’s circle of care provided an overview of the study and obtained consent for research staff to approach the patient. Research personnel obtained written informed consent and administered baseline questionnaires on-site. Physiological variables [fasting blood glucose concentration (mmol/L), glycated hemoglobin (HbA1c;%), HDL cholesterol ratios] were assessed at baseline by CR staff as part of standard intake procedure and then again at 3 months when participants returned for on-site follow-up appointments as part of standard care at the CR program. With respect to psychosocial variables, anxiety, depression, and quality of life were assessed at baseline and 3 months, while attachment and traumatic stress were assessed at baseline only. For those variables measured at 3 months, follow-up questionnaires (3 months) were mailed to participants along with a prepaid return envelope so that participants could mail back completed questionnaires.

### Measures

#### Demographic and Clinical Information

At baseline, participants reported their age, sex, ethnicity, and marital status (see Table 1). Compliance with CR was calculated as the percentage of prescribed CR appointments patients attended. Participants’ cardiac diagnoses and relevant cardiac procedures are also provided in Table 1.

**TABLE 1:** Demographic characteristics of sample.

*N* = 201		*n* (%)
Sex	Male	160 (79.6)
	Female	41 (20.4)
Marital Status	Single	23 (11.4)
	Married/Common Law	150 (74.6)
	Divorced/Separated	15 (7.5)
	Widowed	13 (6.5)
Race	Caucasian	189 (94.0)
	Black	3 (1.5)
	Other	9 (4.5)
Education	Less than High School	23 (11.4)
	High School	47 (23.4)
	University	131 (65.2)
Cardiac Diagnoses or Event	Angina	56 (27.9)
	Heart Failure	22 (11)
	Congenital Heart Problem	6 (3.0)
	Myocardial Infarction	88 (43.8)
	Valvular Heart Disease	29 (14.4)
Cardiac Procedure	PCI	97 (48.3)
	ICD/Pacemaker	8 (4.0)
	Transplant	1 (0.5)
	Valve Procedure	3 (1.5)
	CABG	53 (26.4)
	None	39 (19.4)

*CABG, coronary artery bypass grafting; PCI, percutaneous coronary intervention; ICD, implantable cardioverter defibrillator.*

#### Experiences in Close Relationships-Revised

Attachment style was measured using the validated (Fraley et al., 2000) and reliable (Sibley et al., 2005) Experiences in Close Relationships-Revised (ECR-R). The ECR-R has 36 items designed to measure two individual factors of attachment: attachment anxiety (i.e., the degree to which a person worries their attachment figures will not be available or supportive when they need them) and attachment avoidance (i.e., the degree to which a person distrusts their attachment figures and achieves self-assurance by maintaining emotional distance). Each item consists of a statement regarding how the respondent feels in emotionally intimate relationships. Responses are indicated on a 7-point Likert-type scale (1 = *Strongly Disagree*; 7 = *Strongly Agree*) and the summed total of all items in each attachment style represents the score for that attachment style. Higher values indicate stronger affiliation with a particular attachment style.

#### Impact of Event Scale-Revised

Traumatic stress was measured using the validated (Horowitz et al., 1979) and reliable (Rash et al., 2008), 22-item Impact of Event Scale-Revised (IES-R). On the IES-R, respondents are asked questions about how they have been feeling over the last week with reference to their traumatic event (participants were asked specifically to refer to their cardiac event). Responses are indicated on a 5-point Likert-type scale (0 = *Not at All*; 4 = *Extremely*) and the summed total of all items serves as the total score. Higher scores indicate greater traumatic stress and scores of 33 or higher are indicative of a probable diagnosis of PTSD. The IES-R has been previously validated in a CVD population (Eslami, 2017).

#### Anxiety and Depression

The HADS (Zigmond and Snaith, 1983) is a 14-item self-report measure of anxiety (seven items) and depression (seven items) that has been used often in clinical populations, including with cardiac patients (Tulloch et al., 2018; Lemay et al., 2019). The HADS examines a range of anxious (e.g., muscle tension, panic-like symptoms, constantly worrying) and depressive (e.g., decreased mood, loss of interest, lack of motivation) symptoms. Symptoms statements are rated on a scale from 0 to 3. The HADS produces separate total scores for anxiety and depression (scores range from 0 to 21), with higher scores indicating greater levels of anxiety or depression; scores of 5 (depression) and 7 (anxiety) or greater are considered the cut-offs for clinical screening (Singer et al., 2009). The validity (Bjelland et al., 2002) and reliability (Boxley et al., 2016) of this measure has been demonstrated.

#### Quality of Life

Physical and mental quality of life was measured using the Short Form Health Survey Questionnaire, Version 1 (SF-36, V.1; Ware and Sherbourne, 1992), a self-report questionnaire containing 36 items. For the physical health (i.e., summed total of scale scores for “physical functioning,” “physical role functioning,” “bodily pain,” and “general health perceptions”) and mental health (i.e., summed total of scale scores for “vitality,” “emotional role functioning,” “social role functioning,” and “mental health”) total scores, higher values indicate better perceived physical or mental quality of life, respectively. This measure has been validated previously in a CVD population (Muller-Nordhorn, 2004) and has demonstrated reliability (Bunevicius, 2017).

#### Physiological Health Outcomes

Blood samples were drawn to assess physiological health outcomes, including fasting blood glucose concentration (mmol/L), glycated hemoglobin (HbA1c;%), and cholesterol information, including HDL ratios (total cholesterol/HDL), which have been shown to be superior predictors of poor cardiovascular health than other indicators of cholesterol (Lemieux et al., 2001); higher cholesterol is a risk factor for CVD as it indicates greater risk of arterial plaque deposits. Greater fasting blood glucose or HbA1c are indicative or greater risk of CVD, and can indicate prediabetes or diabetes.

### Data Analyses

**FIGURE 1:**
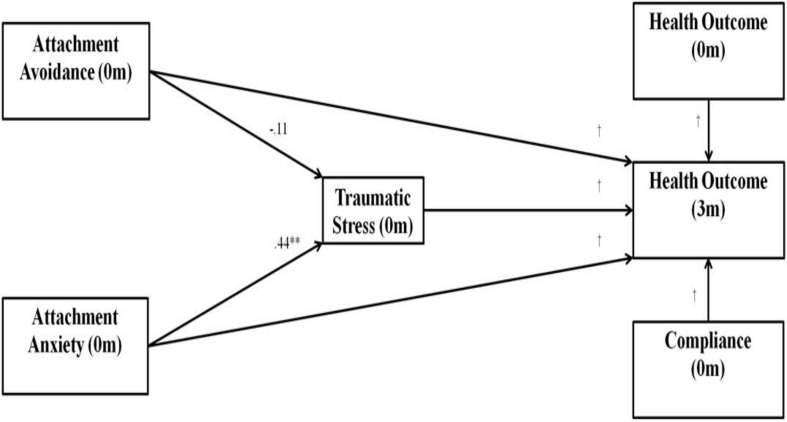
Multivariate structural equation model used for path analysis. Hypothesized pathways included attachment anxiety and avoidance having indirect effects on health outcome variables through traumatic stress. m, months.

Missing data were evaluated following the procedures outlined by Tabachnick and Fidell (2007), and were imputed using multiple imputation (i.e., missing values replaced with means of 5 repeated imputations; Rubin, 1996). The effect of participating in the CR program on all dependent variables was assessed using a repeated-measures MANOVA. For the multivariate SEM analyses, the following parameters were used to determine excellent model fit: non-significant χ^2^, CFI ≥ 0.95, RMSEA < 0.07, and values ranging from 2 to 3 for CMIN/df (Hooper et al., 2008). Modification indices were examined to identify instances where variables and error terms were highly correlated; these relationships were allowed to covary within the model to improve fit. The main theoretical model consisted of baseline attachment anxiety and attachment avoidance, predicting traumatic stress, with traumatic stress in turn predicting all end-of-intervention health outcomes (see Figure 1). Compliance and baseline measures of health outcome variables were included in the model as controls. For all standardized indirect coefficients, the bias-corrected percentile method of generating two tailed significance values was used. Indirect effects were calculated using 2000 bootstrap samples.

## Results

### Preliminary Analyses

All variables were normally distributed. For baseline data, 9 and 10% of the data were missing for attachment anxiety and attachment avoidance subscales respectively, while 8% of the data were missing for the IES-R. Regarding information collected at baseline and 3 months, HADS scores were missing 1 and 23%, respectively, SF-36 scores were missing 0 and 33%, fasting blood glucose values were missing 2 and 21%, glycated hemoglobin (HbA1c) values were missing 4 and 29%, and HDL ratio values were missing 1 and 20%. Compliance data were missing 10%. Missing values were found to be missing at random, Little’s MCAR (747) = 42.87, *p* = 0.999.

### Sample Characteristics

We approached 410 patients for the study. Of those, 409 were eligible (1 was not eligible due to language barrier), 288 agreed to participate (70% of eligible patients), and 201 completed the baseline questionnaires (70% of consented patients). Between baseline and follow-up, 2 patients died, 2 were out of the country, 9 dropped out of the study (2 said they felt it was not relevant to them, 2 had insufficient time, and 5 stated they were no longer interested), and 23 were unreachable, leaving 165 participants (82%) with completed 3-month data.

A demographic summary is presented in Table 1. The participants (*n* = 201) ranged in age from 24 to 89 years (*M* = 62.08, *SD* = 10.87) and were predominately male (*n* = 160, 80%), white (*n* = 189, 94%), and married (*n* = 150, 75%). Regarding traumatic stress, 42 (21%) scored above 33 on the IES-R, suggesting the probable presence of PTSD. On the HADS at baseline, a minority of individuals with scores above the cut-offs on anxiety (*n* = 86, 43%) and depression (*n* = 75, 37%); at 3 months, the number of those who scored high on the anxiety subscale had dropped moderately (*n* = 55, 27%) while those above the depression cut-off dropped off to a lesser degree (*n* = 61, 30%). The sample mean for fasting blood glucose concentration was above the normal range of 3.9 – 5.6 mmol/L (The Emerging Risk Factors Collaboration, 2010) at both baseline and 3 months and the sample mean for HbA1c was above the normal range of 3.8–5.9% (Cavalot et al., 2006) at both baseline and 3 months. The sample mean for HDL ratio was below the highest risk cut-off of 5 at both baseline (*M* = 3.25, *SD* = 0.96) and 3 months (*M* = 2.99, *SD* = 0.79) (Manninen et al., 1992). Compliance was high (*M* = 82.42%, *SD* = 25.17); about a third of participants attended every session (*n* = 73, 36.3%).

### Effects of the Cardiac Rehabilitation Program

A repeated measures MANOVA on all dependent variables was significant, *F*(7,194) = 29.78, *p* < 0.001, Wilk’s λ = 0.48, partial η^2^ = 0.52. Depression (*p* = 0.003), anxiety (*p* < 0.001), and HDL ratio (*p* < 0.001), were all significantly lower at 3 months compared to baseline; physical quality of life (*p* < 0.001) and mental quality of life (*p* < 0.001) were significantly higher at 3 months compared to baseline. There was no significant change in fasting blood glucose or HbA1c values between baseline and 3 months. See Table 2 for all means, standard deviations, and zero-order correlations between variables.

**TABLE 2:** Means, standard deviations, and correlations for all study variables.

	Variables	*M* (*SD*)	1	2	3	4	5	6	7	8	9	10	11	12	13	14	15	16	17
1	IES-R (0m)	20.03 (16.79)	–																
2	ECR-Avd (0 m)	44.63 (20.64)	0.14*	–															
3	ECR-Anx (0 m)	36.93 (16.82)	0.43**	0.52**	–														
4	HADS-Anx (0 m)	5.98 (3.91)	0.57**	0.22**	0.39**	–													
5	HADS-Dep (0 m)	3.91 (3.09)	0.48**	0.25**	0.40**	0.56**	–												
6	HADS-Anx (3 m)	4.68 (3.47)	0.52**	0.21**	0.35**	0.66**	0.42**	–											
7	HADS-Dep (3 m)	3.36 (3.09)	0.46**	0.25**	0.38**	0.43	0.66**	0.60**	–										
8	SF-36 Phys (0 m)	52.71 (20.75)	−0.29**	–0.13	−0.20**	−0.32**	−0.52**	−0.24**	−0.35**	–									
9	SF-36 Ment (0 m)	61.31 (20.86)	−0.48**	−0.26**	−0.39**	−0.56**	−0.64**	−0.35**	−0.48**	0.65**	–								
10	SF-36 Phys (3 m)	67.65 (22.69)	−0.34**	−0.17*	−0.22**	−0.43**	−0.55**	−0.47**	−0.64**	0.65**	0.45**	–							
11	SF-36 Ment (3 m)	72.82 (20.15)	−0.50**	−0.31**	−0.44**	−0.54**	−0.62**	−0.71**	−0.79**	0.45**	0.56**	0.74**	–						
12	FBG (0 m)	5.76 (1.28)	0.04	0.03	0.08	–0.02	0.06	–0.01	0.09	−0.24**	–0.06	−0.16*	–0.08	–					
13	FBG (3 m)	5.83 (1.31)	0.16*	0.12	0.09	0.01	0.09	0.03	0.15*	−0.26**	–0.08	−0.21**	−0.14*	0.82**	–				
14	HbA1c (0 m)	6.04 (0.88)	–0.03	0.13	0.02	–0.10	0.02	–0.04	0.03	–0.09	0.03	–0.04	–0.03	0.57**	0.69**	–			
15	HbA1c (3 m)	6.06 (0.76)	0.12	0.09	–0.01	–0.11	0.03	–0.06	0.05	−0.20**	–0.02	–0.13	–0.11	0.69**	0.84**	0.80**	–		
16	HDL Ratio (0 m)	3.25 (0.96)	–0.05	0.04	0.08	–0.01	0.12	0.03	0.15*	–0.08	–0.08	−0.14*	−0.14*	0.09	0.12	–0.02	0.06	–	
17	HDL Ratio (3 m)	2.99 (0.79)	–0.03	0.01	0.14*	0.01	0.07	0.06	0.09	–0.02	–0.05	–0.09	–0.13	0.15*	0.26**	0.11	0.21**	0.74**	
18	Compliance	82.43 (25.17)	–0.02	–0.02	0.04	0.09	0.11	0.11	0.13	−0.15*	−0.14*	−0.16*	–0.13	0.13	0.00	−0.18*	–0.05	–0.01	–0.09

*p < 0.05, **p < 0.01, *M*, means; *SD*, standard deviation; m, months; Avd, avoidance subscale; Anx, anxious subscale; Dep, depression subscale; Phys, physical health subscale; Ment, mental health subscale; FBG, fasting blood glucose.

### Structural Equation Modeling

**FIGURE 2:**
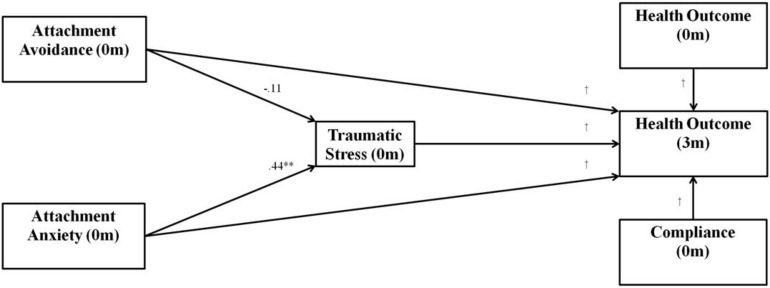
Multivariate structural equation model used for path analysis with values representing standardized regression coefficients. The hypothesized pathways include attachment anxiety and avoidance having indirect effects on health outcome variables through traumatic stress. ^∗∗^*p* < 0.01, m, months, ^†^values are presented in Table 2. Indirect effects for model are shown in Table 3.

The multivariate SEM model (with standardized estimates) can be found in Figure 2. The model fit statistics indicated the hypothesized model was a good fit of the data; the chi-square test was not significant [χ^2^(74) = 91.30, *p* = 0.084], and the measures of approximate fit were: RMSEA = 0.03 (CI_90__%_ = 0.00,0.06), CFI = 0.99, CMIN/df = 1.23.

The results of the path analysis were that baseline attachment anxiety had a significant, positive indirect effect (via traumatic stress) on depression, anxiety, fasting blood glucose, and HbA1c, and a significant, negative indirect effect on mental quality of life and physical quality of life (see direct effects in Table 3 and indirect effects in Table 4). The indirect effect of baseline attachment anxiety on HDL ratio was not significant; however, greater baseline attachment anxiety directly predicted significantly greater HDL ratios. There were no significant indirect effects of baseline attachment avoidance on any outcome variable.

**TABLE 3:** Standardized direct effects and *R*^2^ values for multivariate structural equation model.

			Direct Effects on Outcome Measures (3 m)				
		Attachment Avoidance (0 m)	Attachment Anxiety (0 m)	Traumatic Stress (0 m)	Baseline Health Outcome (0 m)	Compliance (0 m)	*R*^2^
Psychosocial Measures	HADS Anxiety	0.04	0.05	0.23**	0.46**	0.08	0.44
	HADS Depression	0.07	0.08	0.23**	0.45**	0.09	0.44
	SF-36 Physical QOL	–0.04	0.02	−0.13*	0.60**	–0.08	0.40
	SF-36 Mental QOL	–0.07	−0.17*	−0.16*	0.32**	–0.09	0.31
Physical Health Measures	FBG	0.12*	–0.05	0.11**	0.82**	−0.10*	0.68
	HbA1c	0.02	−0.12*	0.14**	0.81**	0.10	0.66
	HDL Ratio	–0.08	0.14*	–0.05	0.72**	–0.08	0.55

*p < 0.05, **p < 0.01, m, months; HADS, Hospital Anxiety and Depression Scale; SF-36, Short Form Health Survey – 36 Item Form; FBG, Fasting Blood Glucose; HbA1c, Glycated Hemoglobin; HDL, High-density Lipoprotein.

**TABLE 4:** Standardized indirect effects for multivariate structural equation model.

		Indirect Effects on Outcome Measures (3 m) via Traumatic Stress	
		Attachment Avoidance (0 m)	Attachment Anxiety (0 m)
Psychosocial Measures	HADS Anxiety	–0.02	0.10**
	HADS Depression	–0.02	0.10**
	SF-36 Physical QOL	0.01	−0.06*
	SF-36 Mental QOL	0.02	−0.07*
Physical Health Measures	Fasting Blood Glucose	–0.01	0.05**
	HbA1c	–0.02	0.06**
	HDL Ratio	0.01	–0.02

*p < 0.05, **p < 0.01, m, months; HADS, Hospital Anxiety and Depression Scale; SF-36, Short Form Health Survey – 36 Item Form; HbA1c, Glycated Hemoglobin; HDL, High-density Lipoprotein.

## Discussion

The purpose of the present study was to test a model predicting physical and mental health outcomes in CVD patients using measures of attachment style and traumatic stress. To this end, CR patients were assessed at baseline (program intake) and 3-month follow-up (end-of-program) on a variety of outcomes.

### Hypothesis 1

The first hypothesis was that insecure attachment (i.e., attachment anxiety or avoidance) would be positively associated with traumatic stress. In support of this, the zero-order correlations (see Table 2) between attachment anxiety, attachment avoidance, and traumatic stress were all significant and positive. The SEM results, however, supported this hypothesis only for attachment anxiety; attachment avoidance did not significantly predict traumatic stress (see Figure 2).

#### Attachment Anxiety and Trauma

One reason why attachment anxiety might associate with greater traumatic stress is that individuals with anxious attachment appear to be more sensitive to detecting threatening stimuli (Barazzone et al., 2019). This focus on potential threat, coupled with generally less perceived social support compared to securely attached individuals, may make those with anxious attachment less resilient to trauma. Perceived social support is known to be among the most important buffers against symptoms of PTSD (e.g., Guay et al., 2006; Stanley et al., 2018). It follows logically that insecurely attached people would be more susceptible to the effects of trauma than would securely attached people.

It has also been proposed that traumatic experiences, such as an acute cardiac event, activate the attachment system. Although both securely attached and anxiously attached individuals are likely to seek out reassurance and comfort from their attachment figures, those who are anxiously attached are less likely to perceive and to integrate the emotional support that is offered. This drives them to persist in their bids for reassurance, which might strain their close relationships and add additional barriers against processing trauma effectively (Mikulincer et al., 2007, 2015).

#### Attachment Avoidance and Trauma

Alternatively, our finding that attachment avoidance did not significantly predict traumatic stress is not without precedent. For example, in one meta-analysis, there was no relationship between attachment avoidance and traumatic stress, though the authors noted that this may have been due to the tendency to underreport, especially when completing self-report measures (Woodhouse et al., 2015). Psychophysiological measures that tap into adrenocortical activity, skin conductance, and heart rate, are generally more sensitive indicators of stress among people with avoidant attachment styles (Gander and Buchheim, 2015). For this reason, it is likely that the lack of a link between attachment avoidance and traumatic stress may be more related to a lack of reporting than to true resilience against trauma. Individuals with an avoidant attachment style tend to dismiss or compartmentalize trauma without seeking support from others, which seems to disrupt the ability to process trauma effectively (Mikulincer et al., 2007). In addition, there is some evidence that individuals with avoidant attachment express fewer positive and negative psychological symptoms, tending to experience psychological symptoms that are negative to neutral (Kerr et al., 2003). In accordance with this, almost all zero-order correlations in the present study between attachment scores and outcome variables were greater for anxious attachment than they were for avoidant attachment. Future research is required to fully understand the relationships between trauma and both types of insecure attachment; however, it is possible our theoretical model is only appropriate for those with anxious attachment.

### Hypothesis 2

Second, it was hypothesized that insecure attachment (i.e., greater attachment anxiety and avoidance) and greater traumatic stress would each independently predict poorer health outcomes. Again, this hypothesis was partially supported (see Table 3).

#### Attachment Anxiety, Attachment Avoidance, and Health

While greater attachment anxiety directly predicted several poorer health outcomes, attachment avoidance predicted significantly higher fasting blood glucose values only. This, as well as the finding that attachment anxiety had significant indirect effects on many outcome variables and attachment avoidance had none (see Table 4), suggests that attachment anxiety, not avoidance, may drive the previously observed relationship between insecure attachment and poorer health (Maunder and Hunter, 2001).

### Hypothesis 3

The third hypothesis was that traumatic stress would mediate the relationship between insecure attachment and poorer physical and mental health outcomes. The results of the multivariate SEM supported this hypothesis for attachment anxiety only.

#### Traumatic Stress as a Mediator of the Effects of Attachment on Physical and Mental Health Outcomes

Greater baseline attachment anxiety was indirectly related to greater anxiety, depression, fasting blood glucose, and HbA1c levels at 3 months, and poorer physical and mental quality of life at 3 months. There were no significant indirect effects of attachment avoidance on any outcome measure; however, our findings are the first evidence that attachment anxiety is associated with poorer health outcomes in CVD patients via the experience of traumatic stress, which is an important finding. It is the first substantial empirical evidence of the notion that people with an anxious attachment style may be so preoccupied with their attachment relationships that it puts their health at risk.

#### Attachment Anxiety

In previous research, attachment anxiety has been linked to greater cortisol levels in times of stress (Quirin et al., 2008), and augmented diastolic and systolic blood pressure during social interactions (Gallo and Matthews, 2006). Our findings complement these and demonstrate that attachment anxiety is highly predictive of negative outcomes in people with heart disease. People with an anxious attachment style experience a great deal of interpersonal stress throughout most of their lives (Simpson and Rholes, 2017); their intense fear of losing meaningful attachment relationships and the accompanying hyperactivation of the attachment system might reach chronic levels similar to the hypervigilance that characterizes PTSD, with all of the health risks this can pose.

#### Attachment Avoidance

Although attachment anxiety was directly and indirectly linked to poor physical and mental health outcomes, attachment avoidance was not. The only finding related to CVD risk factors that was observed for attachment avoidance was that greater attachment avoidance at baseline was directly related to greater fasting blood glucose levels at 3 months. Regarding relationships with mediating factors in the SEM model, greater attachment avoidance predicted significantly lower levels of traumatic stress, which is at odds with previous findings (Clark and Owens, 2012). One possibility is that attachment avoidance may provide some measure of protection or resiliency against traumatic stress, but at the cost of fewer people who could potentially provide positive support. Alternatively, those who score higher in attachment avoidance tend to have less insight into and ability to express their own emotional experiences, otherwise known as alexithymia (Fantini-Hauwel et al., 2012). They might underreport traumatic stress, which may be the more plausible interpretation of this finding.

### Limitations

The present study is not without limitations. Our CR patients were diverse in terms of cardiac diagnoses (see Table 1), meaning our relatively small sample size (*N* = 201) precluded diagnosis-based analyses of our data. Furthermore, we lacked some important variables (e.g., length of time since cardiac event, duration of hospitalization, etc.) that are important in the context of understanding the nature of traumatic stress. In many ways, our sample may be considered a relatively small subsample of the overall cardiac population; larger cohort studies could provide additional evidence regarding the relationship between attachment style and traumatic stress in specific cardiac subgroups. Understanding whether there are differences in terms of cardiac diagnoses would inform screening and treatment considerations in these groups.

Although diverse in terms of CVD, our sample was quite homogenous in other ways, further limiting the degree to which our findings may generalize to other settings. Our group of participants were mainly white, married, and highly educated; while this reflects the nature of who attends CR in our clinical setting and thus has clinical validity here, it makes examining relationships between study variables and sample variables (e.g., ethnicity) very difficult with a sample this size, disqualifying some potentially interesting analyses.

Finally, future studies could conduct longer-term follow up (i.e., compared to the 3 months presented here), and more thorough data collection (e.g., length of time since cardiac event, as listed above). The design of the present study was made with the structure of the current CR program in mind; alternative studies in a less clinical setting could prioritize more rigorous follow-up procedures, longer-term data-collection window, and greater control over the type of data collected from participants.

## Conclusion

In the present sample of patients participating in a CR program, greater attachment anxiety predicted greater traumatic stress; this, in turn, predicted poorer physical and mental health outcomes. Via traumatic stress, baseline attachment anxiety was indirectly linked with a host of negative outcomes at 3 months, including greater anxiety, depression, fasting blood glucose, and HbA1c levels. Stated differently, people who tend to fear emotional abandonment and loss appear to be more susceptible to traumatic stress in the context of heart disease, which in turn seems to have negative effects on their mental and physical health, itself known to further worsen CVD (e.g., Williams and Steptoe, 2007).

Screening and interventions designed to target elevated attachment anxiety and traumatic stress should be considered for future study. Attachment-focused therapies such as Emotionally Focused Individual Therapy (EFIT; Johnson, 2019), Emotionally Focused Therapy (EFT) for couples (Johnson, 2020), or interpersonal psychodynamic approaches (Yalom, 1995) are interventions that might be particularly effective with the CVD population, due to their emphasis on helping clients develop and maintain secure attachment bonds to the most significant people in their lives. Another program that has shown promise among people with heart disease and their partner is Healing Hearts Together (Tulloch et al., 2016, 2017), which is heavily based on the principles of attachment theory and EFT. Whatever the intervention, our findings suggest that a key component ought to be the strength and quality of the personal relationships of patients with heart disease.

## Data Availability Statement

The datasets generated for this study are available on request to the corresponding author.

## Ethics Statement

The studies involving human participants were reviewed and approved by the Ottawa Health Science Network Research Ethics Board. The patients/participants provided their written informed consent to participate in this study.

## Author Contributions

All authors contributed to the writing and conceptualization of the manuscript. AH and HT completed the statistical analyses.

## Conflict of Interest

The authors declare that the research was conducted in the absence of any commercial or financial relationships that could be construed as a potential conflict of interest.

## Abbreviations

CABG: coronary artery bypass grafting
CFI: Comparative Fit Index
CR: cardiac rehabilitation
CVD: cardiovascular disease
ECR-R: Experiences in Close Relationships – Revised
HADS: Hospital Anxiety and Depression Scale
HbA1c: glycated hemoglobin
HDL: high-density lipoprotein
ICD: implantable cardioverter defibrillator
IES-R: Impact of Event Scale – Revised
IL-6: cytokine interleukin 6
MANOVA: multivariate analysis of variance
MCAR: Missing Completely at Random
PCI: percutaneous coronary intervention
PTSD: posttraumatic stress disorder
RMSEA: root mean square error of approximation
SEM: structural equation modeling
SF-36: 36-Item Short Form Survey
UOHI: University of Ottawa Heart Institute.
